# Supplementary figures and images for: Genomic Diversity and Selection Signatures for Zaosheng Cattle
Source: Biology (Basel). 2025 May 28;14(6):623. doi: 10.3390/biology14060623 (PMC12189096; doi:10.3390/biology14060623)

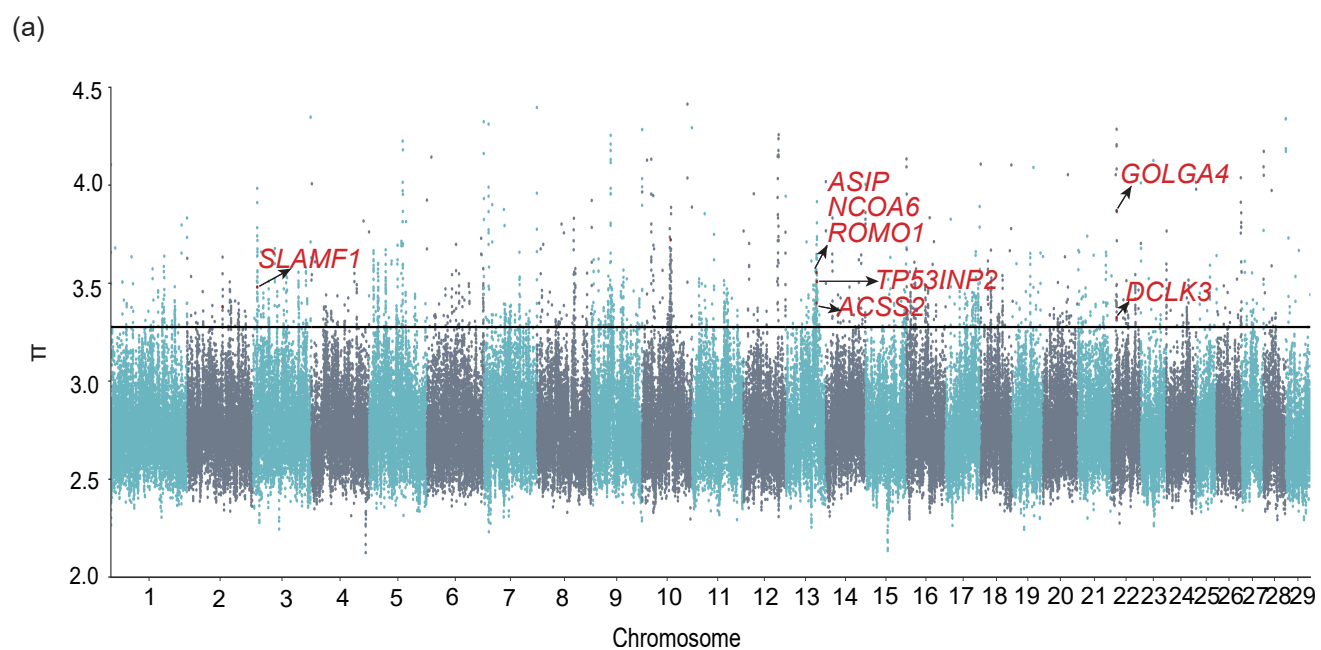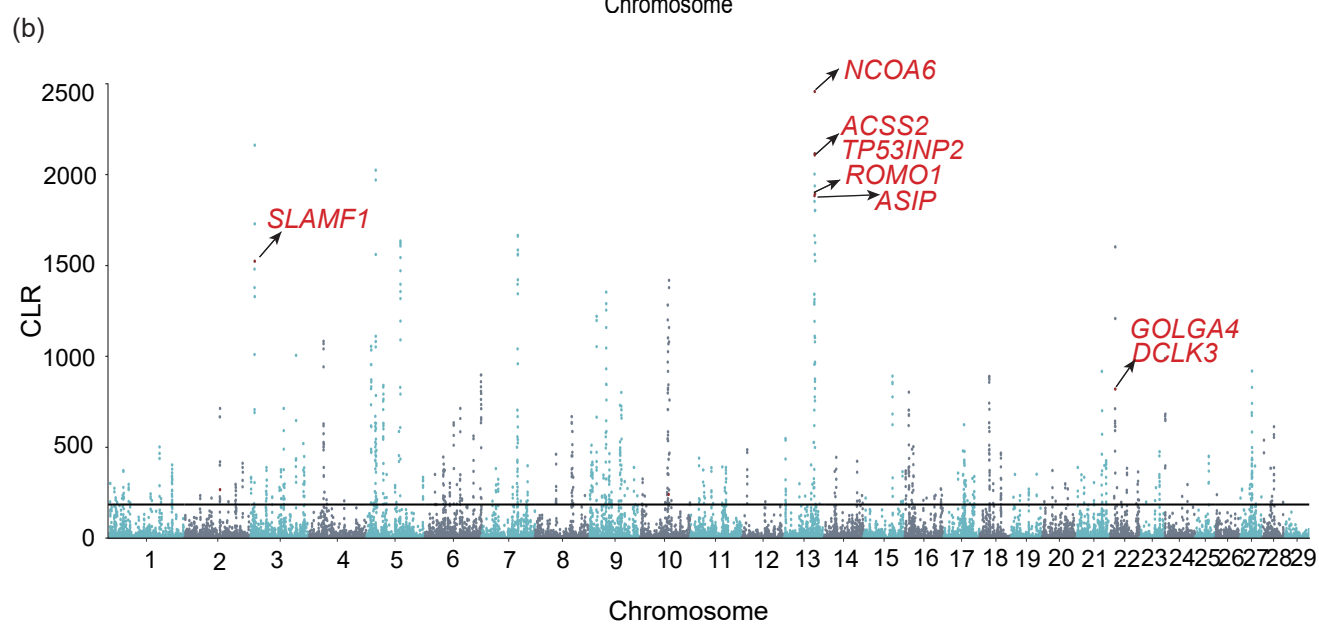

Supplement: Supplementary file 1 [file biology-14-00623-s001.zip › Supplementary materials/Supplementary Figure S1.pdf]
